# Glycobiology of Eosinophilic Inflammation: Contributions of Siglecs, Glycans, and Other Glycan-Binding Proteins

**DOI:** 10.3389/fmed.2017.00116

**Published:** 2017-08-02

**Authors:** Jeremy A. O’Sullivan, Daniela J. Carroll, Bruce S. Bochner

**Affiliations:** ^1^Division of Allergy and Immunology, Department of Medicine, Northwestern University Feinberg School of Medicine, Chicago, IL, United States

**Keywords:** eosinophils, Siglec-8, Siglec-F, selectins, galectins, glycans

## Abstract

The historical focus on protein–protein interactions in biological systems, at the expense of attention given to interactions between other classes of molecules, has overlooked important and clinically relevant processes and points of potential clinical intervention. For example, the significance of protein–carbohydrate interactions, especially in the regulation of immune responses, has recently received greater recognition and appreciation. This review discusses several ways by which cell-surface lectin–glycan interactions can modulate eosinophil function, particularly at the levels of eosinophil recruitment and survival, and how such interactions can be exploited therapeutically. A primary focus is on discoveries concerning Siglec-8, a glycan-binding protein selectively expressed on human eosinophils, and its closest functional paralog in the mouse, Siglec-F. Recent advances in the synthesis of polymeric ligands, the identification of physiological ligands for Siglec-8 and Siglec-F in the airway, and the determination of the basis of glycan ligand discrimination of Siglec-8 are discussed. Important similarities and differences between these siglecs are outlined. Eosinophil expression of additional glycan-binding proteins or their glycan ligands, including interactions involving members of the selectin, galectin, and siglec families, is summarized. The roles of these molecules in eosinophil recruitment, survival, and inflammation are described. Finally, the modulation of these interactions and potential therapeutic exploitation of glycan-binding proteins and their ligands to ameliorate eosinophil-associated diseases are considered.

Eosinophils are innate immune cells that contribute to host defense responses against parasitic infections and appear to have been retained in evolution throughout vertebrate species (1–3). Yet, there is a sizable body of evidence that eosinophils, under other circumstances, can be pro-inflammatory and are thus thought to be major effector cells in allergic and other type 2 immune responses. These include common conditions such as asthma, often manifesting with comorbid upper airways diseases of chronic rhinosinusitis with or without nasal polyposis, disorders that similarly manifest prominent type 2 inflammatory signatures and features including elevated Th2 and ILC2 cells with IL-4, IL-5, IL-13, eotaxins, and other downstream mediators (4–7). Another common eosinophil-associated disease is atopic dermatitis, where eosinophils contribute to some but perhaps not all stages of the disease (8). Less common disorders where eosinophils are felt to play a major role include eosinophilic granulomatosis with polyangiitis (formerly known as Churg–Strauss syndrome); eosinophilic gastrointestinal disorders (EGID), namely eosinophilic esophagitis, gastritis, and colitis, existing alone or in combination (9–13); and other systemic and organ-specific hypereosinophilic syndromes and disorders (14).

Current treatments for these eosinophil-associated conditions include glucocorticosteroids, mediator receptor antagonists, and other anti-inflammatory drugs that reduce eosinophil numbers and activity, but they are neither fully effective nor curative or disease modifying, hence the need for additional therapies (15, 16). Advanced efforts to indirectly target eosinophils [e.g., with agents that antagonize TSLP (17) and IL-4 and IL-13 biology with FDA-approved dupilumab (18–21)] or more specifically target eosinophils (e.g., with the FDA-approved anti-IL-5 biologics mepolizumab and reslizumab, and perhaps someday with the anti-IL-5 receptor antibody benralizumab and the oral agent dexpramipexole) offer hope for improved management of these disorders (22–24). Despite these promising agents, and advancements in our understanding of the pathophysiology of each of these disorders, many patients remain refractory to treatment, or in the case of EGID, there are as yet no FDA-approved drugs. These and other unmet needs led to collaborative efforts to find additional eosinophil-selective targets, and in recent years, these have included the only known pure eosinophil-specific surface target EMR1 (25, 26), and Siglec-8, expressed not only on eosinophils but also on mast cells and weakly on basophils (27–29). The focus of this review is not only on the latter molecule but also includes discussions of other lectin–glycan interactions known to influence eosinophil responses.

## Siglec-8

### Receptor Discovery, Characteristics, and Expression Patterns

Siglec-8 [also originally named sialoadhesin family 2 (SAF-2)] is an I-type single pass transmembrane protein that was discovered from a human eosinophil cDNA library generated from a patient with hypereosinophilic syndrome and first described in the year 2000. Eosinophil mRNA was examined by random sequencing of expressed sequence tags, which led to the identification of a protein 431 amino acid residues (aa) in length that was highly homologous to others in the sialoadhesin/siglec family. The highest levels of homology were found with Siglec-7 (68%), Siglec-3 (49%), and Siglec-5 (42%). The extracellular region of Siglec-8 contains 358 aa with a hydrophobic signal peptide and three Ig-like domains, with the N-terminal Ig domain possessing an arginine at position 125 that is putatively necessary for sialic acid binding (27, 28). When originally described, the cytoplasmic domain was found to be unusually short, and no known signaling motifs were observed. Subsequent investigations by Foussias et al. led to the observation that Siglec-8 exists in two isoforms (the 431-aa originally identified Siglec-8 “short form” and a 499-aa Siglec-8 “long form”), both containing identical extracellular and transmembrane regions. However, like most other CD33-related siglecs, the long form of Siglec-8 contained two characteristic tyrosine-based motifs: a membrane-proximal immunoreceptor tyrosine-based inhibitory motif (ITIM) resembling a classical ITIM (ILVxYxxLV) and a membrane-distal immunoreceptor tyrosine-based switch motif (ITSM) resembling a motif (TxYxxIV) found in the intracellular region of signaling lymphocyte activation molecule (SLAM) (30). The Siglec-8 long form is now just called Siglec-8 because it was found to be the primary form of the receptor, with a molecular weight of ~54 kDa (30), although eosinophils usually but not always express the short form, the function of which remains unknown (31).

While quantitative PCR analysis for the Siglec-8 mRNA not surprisingly detected signals in hematopoietic organs, expression in lung was unexpected. Using monoclonal antibodies recognizing the extracellular region, it was soon discovered that Siglec-8 was not just an eosinophil marker. It was selectively expressed on the surface of eosinophils, mast cells, and at low levels on basophils, but not on any other cells, making it the first receptor to be exclusively expressed on these three allergic effector cell types (28). Using human CD34+ cell-derived culture systems, it was determined that Siglec-8 is a terminal differentiation marker in both eosinophils and mast cells, with maximum protein expression in each cell type occurring at 21 and 30 days of culture, respectively. In contrast, none of the eosinophilic cell lines express Siglec-8 and only modest expression was observed on the mast cell line HMC-1.2, furthering the concept that Siglec-8 is a terminal differentiation marker on these cell types (32, 33).

The *SIGLEC8* gene, like other CD33-related siglecs, is located in the centromeric region of chromosome 19q13 (27, 30). However, little is known about regulation of *SIGLEC8* expression at the transcriptional level. In a recent report, Hwang et al. identified Olig2, a basic helix-loop-helix transcription factor, as a potential regulator of *SIGLEC8* gene expression. They showed that *OLIG2* and *SIGLEC8* are coexpressed late in eosinophil differentiation and that both proteins are expressed in terminally differentiated eosinophils. Furthermore, they showed that Olig2 siRNA reduced *SIGLEC8* mRNA and Siglec-8 surface protein levels, suggesting that Olig2 is a transcriptional regulator of the *SIGLEC8* gene (34). However, all of the currently available human eosinophilic cell lines express Olig2 protein but fail to express Siglec-8, as noted earlier. In addition, Olig2 is not expressed in cord blood-derived mast cells that express Siglec-8. Thus, it appears that *SIGLEC8* gene expression is only partially regulated by Olig2 and further work is needed to determine the exact combination of transcription factors responsible for Siglec-8 expression (33, 34).

### Ligands for Siglec-8

**Figure 1:**
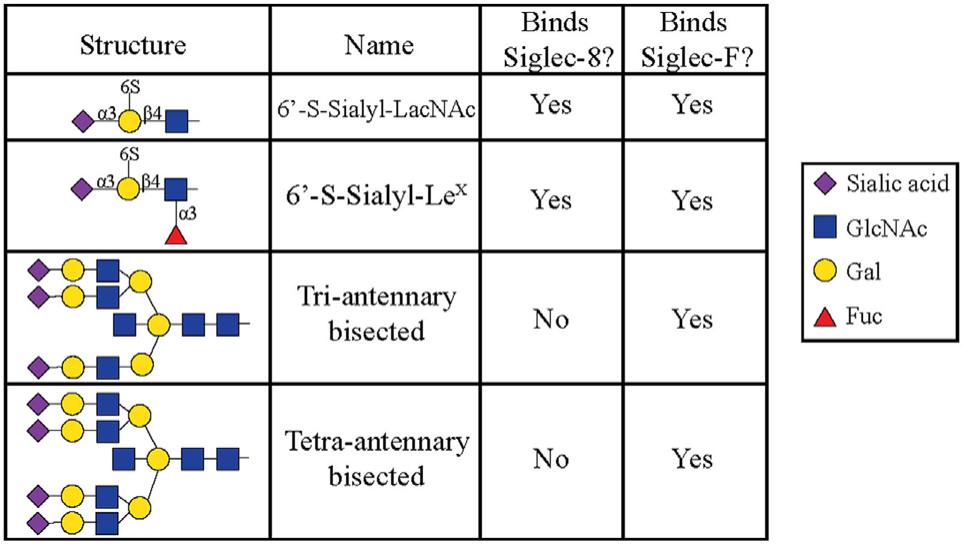
Glycans recognized by Siglec-8-Fc and Siglec-F-Fc, as determined by using glycan microarray analysis, are shown. Reproduced and modified from Ref. (38) with permission.

All siglecs contain an amino-terminal V-set Ig lectin domain that binds sialic acid, but each siglec has a characteristic specificity profile for binding only certain conformations of sialic acid. Most siglecs recognize α2-3- and α2-6-linked sialic acids, although some can also recognize α2-8-linked sialic acids (35, 36). Initial experiments to characterize Siglec-8 ligand-binding preferences demonstrated that Siglec-8 preferentially binds α2-3-sialic acids linked to Galβ1-4GlcNAc (27). Using a glycan array generated by the Consortium for Functional Glycomics, 172 glycan structures were screened, and it was discovered that Siglec-8 specifically bound 6′ sulfated sialyl Lewis^X^ (6′-sulfo-sLe^x^ or NeuAcα2-3Galβ1-4(Fucα1-3)(6-*O*-Sulfo)GlcNAcβ1). Siglec-8 did not bind sialyl Lewis^X^, a common ligand for L-, P-, and E-selectins, demonstrating that the 6′-position sulfate on the galactose was absolutely necessary for Siglec-8 lectin-glycan binding (37). A subsequent re-screen of an expanded array containing over 600 structures revealed that the fucose was dispensible (38) (Figure 1). Experiments using heparinized whole blood showed that a polyacrylamide polymer decorated with 6′-sulfo-sLe^x^ bound only eosinophils in a Siglec-8-dependent manner, further demonstrating that this glycan is a specific ligand for Siglec-8 (39).

**Figure 2:**
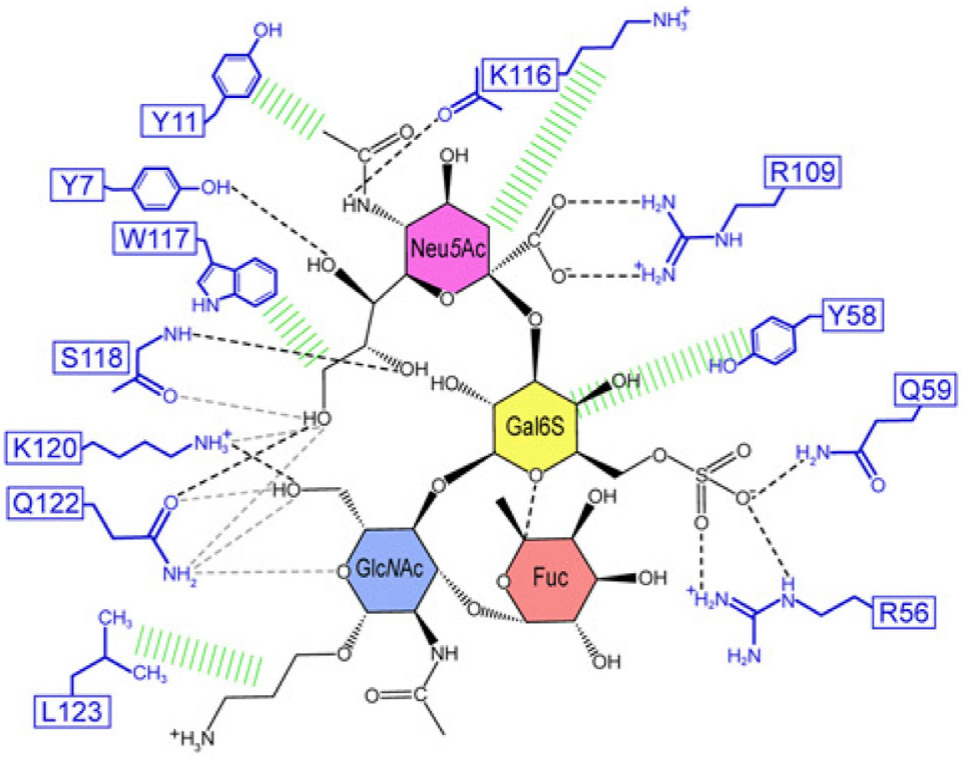
Structural basis for 6′ sulfated sLex recognition by human Siglec-8 illustrated by a representative structure (lowest energy) of the NMR ensemble. Schematic illustration of the Siglec-8–6′ sulfated sLex interaction network. Black dashed lines indicate hydrogen bonds in the depicted structure; gray dashed lines indicate hydrogen bonds abundantly observed in other structures of the ensemble. Hydrophobic contacts are shown in green. Reproduced from Ref. (40) with permission.

The structural basis of how Siglec-8 interacts with its glycan ligand had been unexplored until a recent report by Propster et al., where they provide a detailed description of how Siglec-8 selectively recognizes its ligand, 6′-sulfo-sLe^x^. First, using NMR spectroscopy, they determined the 3D structure of the lectin domain of Siglec-8. The structure is a V-set Ig-like β-sandwich of two antiparallel β-sheets formed by β-strands ABED and C′CFG, with the conserved arginine, responsible for providing a salt bridge interaction with sialic acid, located on β-strand F. Ligand specificity is mediated by two motifs, where the primary motif recognizes the terminal Neu5Ac, similar to other siglecs, and the secondary motif recognizes the subterminal Gal6S, which was found to be unique among siglecs. Although amino acid mutations failed to affect the overall structure of Siglec-8, a mutation in the conserved arginine eliminated Neu5Ac binding and completely abrogated Siglec-8–ligand interactions. In accordance with previous work done in our lab, this group also demonstrates that the sulfate modification was absolutely necessary for enhanced ligand affinity and revealed the key determinants for glycan specificity (40) (Figure 2).

### Siglec-8 Function on Human Eosinophils

Initial clues regarding how Siglec-8 might function came from an examination of its structural motifs. The cytoplasmic domain of Siglec-8 contains one ITIM and one ITSM, thought to be responsible for initiating downstream receptor function. Using peripheral blood human eosinophils, it was shown that multimeric engagement of Siglec-8 with a mouse monoclonal antibody (mAb) causes Siglec-8-dependent eosinophil apoptosis. However, this required the presence of a secondary anti-mouse cross-linking antibody; without secondary antibody, no cell death was seen (41). Further studies to delineate the mechanisms through which Siglec-8 induces eosinophil apoptosis revealed that Siglec-8 cross-linking promoted reactive oxygen species (ROS) production, loss of mitochondrial membrane potential and caspase cleavage (41, 42). Additionally, Siglec-8-dependent eosinophil apoptosis was paradoxically amplified under conditions of eosinophil priming with IL-5, GM-CSF, or IL-33, eliminating the need for secondary cross-linking antibody and changing the apoptotic mechanism to one dependent on ROS rather than caspase activity (41–44). Furthermore, incubation of IL-5-primed eosinophils with a synthetic polyacrylamide polymer decorated with 6′-sulfo-sLe^x^ induced eosinophil apoptosis (39), suggesting that Siglec-8 functions through different mechanisms in the presence or absence of cytokine priming.

In addition to studies using a mAb and a synthetic ligand, von Gunten et al. discovered that exposure of IL-5-primed eosinophils to intravenous immunoglobulin (IVIG), often used at high doses for the treatment of autoimmune and allergic diseases, resulted in eosinophil cytotoxicity. Further experiments revealed that IVIG contained autoantibodies against Siglec-8 that were responsible for this cytokine-dependent apoptotic effect of IVIG and that this effect was ROS-dependent (45), similar to what was observed when using mAbs to Siglec-8.

Although the intracellular signaling pathways for most siglecs are not well characterized, several studies have shown that engagement of CD33-related siglecs leads to downstream activation of Src family kinases (SFKs) that provide docking sites for Src-homology region 2 domain-containing phosphatases such as SHP-1 and SHP-2 that then propagate downstream functions (46–49). Indeed, ongoing work to further explore Siglec-8 signaling on eosinophils revealed that Siglec-8 engagement on IL-5-primed eosinophils leads to phosphorylation of SFKs, and use of SFK pharmacological antagonists inhibited Siglec-8-mediated eosinophil ROS production and apoptosis, although the SFKs involved in Siglec-8 function have yet to be determined (50). Furthermore, preliminary data show that Siglec-8 associates with SHP-2 and that pharmacological inhibition of protein tyrosine phosphatases inhibits Siglec-8-mediated eosinophil apoptosis (51). Together, these studies support the notion that Siglec-8 functions similar to other CD33-related siglecs.

The presence of an ITIM suggests that Siglec-8 should be involved in negative cell signaling; however, some of the latest observations suggest that Siglec-8 can, under certain circumstances, function as an activating receptor, such as after IL-5 priming. Initial evidence supporting this hypothesis showed that Siglec-8 cross-linking leads to enhanced phosphorylation of extracellular signal-regulated kinase (ERK) 1/2 and activation of ERK1/2 was necessary for Siglec-8-mediated eosinophil apoptosis (50).

## Siglec-F

In view of the usefulness of mouse models for functional manipulations, it was imperative to identify a suitable mouse homolog of Siglec-8. In mice, there is no Siglec-8 ortholog, but Siglec-F has been found to be the closest functional paralog. Siglec-F is a 569-aa, CD33-related siglec that contains four Ig-like domains (Siglec-8 contains three) and, like Siglec-8, it contains both ITIM and ITIM-like motifs in its cytoplasmic tail. Using sequence homology comparisons, it was initially proposed that Siglec-F was the likely ortholog of human Siglec-5, but the homology was limited to the extracellular domains of both receptors (47). Furthermore, initial studies revealed that Siglec-F was predominantly expressed in bone marrow cells of the myelomonocytic lineage, and it was not expressed on mature neutrophils and monocytes (47), which have been shown to express Siglec-5 (52), further suggesting that Siglec-F may not be the true ortholog of Siglec-5.

**Figure 3:**
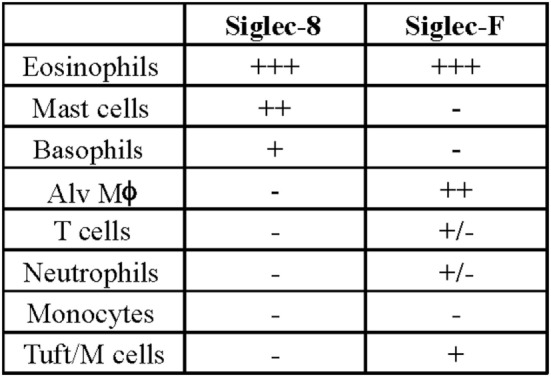
Comparison of cellular surface expression patterns for Siglec-8 and Siglec-F. Alv Mϕ, alveolar macrophage.

Later efforts to fully characterize the expression pattern of Siglec-F and determine its functional counterpart in humans revealed that Siglec-F shared 38% similarity with human Siglec-8 (53). Using monoclonal antibodies to Siglec-F, it was found that Siglec-F, like Siglec-8, was predominantly expressed on the surface of mature eosinophils and on bone marrow eosinophils (54). However, Siglec-F is not expressed on mouse mast cells and surprisingly is instead expressed on mouse alveolar macrophages and subpopulations of intestinal epithelial cells (55–57) (Figure 3). Although they are structurally different and are expressed on different cell types, Siglec-F, like Siglec-8, has a binding preference for α2-3-linked sialic acids and recognizes 6′-sulfo-sLe^x^ (58). While subsequent studies have reproduced these findings, access to additional glycan structures for screening has allowed the identification of several multi-antennary structures that are recognized by Siglec-F but not Siglec-8 (38) (Figure 1). Indeed, this may explain why mouse lung ligands are recognized by Siglec-F but not by Siglec-8 (see below) (59). Based on these reports, it was concluded that Siglec-F and Siglec-8 are functionally convergent paralogs rather than orthologs.

Subsequent studies to examine the biological roles of Siglec-F *in vivo* revealed that its expression is upregulated following allergen challenge in a mouse lung allergy model and the congenital deficiency *via* genetic deletion of the Siglec-F gene led to enhanced eosinophil numbers in the bone marrow, peripheral blood, and lungs during allergic inflammation but not at baseline. Furthermore, Siglec-F-null mice had diminished eosinophil death, suggesting a role for Siglec-F in mediating eosinophil apoptosis (60). Indeed, administration of anti-Siglec-F antibody reduced peripheral blood and tissue eosinophil numbers in wild-type mice, IL-5 transgenic mice, and in mouse models of hypereosinophilic syndrome and eosinophilic esophagitis, which was attributed to induction of eosinophil apoptosis. Additionally, the effect of the anti-Siglec-F antibody was specific to eosinophils and had no effect on other cells, not even Siglec-F-expressing alveolar macrophages (61–64). Despite our advances in understanding the role of Siglec-F in eosinophil survival *in vivo* and *in vitro*, little is known about the signaling mechanism of this receptor. A report by Mao et al. showed that Siglec-F engagement on mouse eosinophils led to caspase cleavage; however, unlike Siglec-8, there was no detectable ROS production, and Siglec-F function did not involve the activation of SFKs or SHP-1 (65). Therefore, further studies are needed to fully characterize the signaling pathways for Siglec-F.

## Tissue Ligands for Siglec-F and Siglec-8

**Figure 4:**
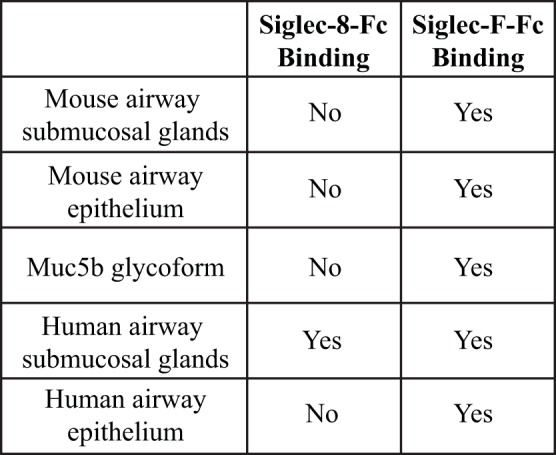
Comparison of binding of Siglec-F and Siglec-8 to mice and human tissue-based sialoside ligands. Based on data from Ref. (59).

Although both Siglec-F and Siglec-8 preferentially recognize the glycan 6′-sulfo-sLe^x^, the identity of their natural ligands is still under investigation. Initial studies to identify endogenous tissue ligands in mice using Siglec-F-Fc chimeras and immunohistochemistry showed that Siglec-F ligands were constitutively expressed on airway epithelial cells and their expression was dependent on ST3Gal-III, a sialyltransferase that can add α2,3 terminal sialic acids to glycans (66). Expression of these ligands was increased upon induction of allergic airway inflammation (67, 68). Glycoproteomic analysis of material derived from mouse tracheal epithelial cells revealed that Siglec-F-Fc bound to glycans displayed on Muc5b and Muc4, but not Muc5ac. Mouse lungs deficient in Muc5b had reduced Siglec-F-Fc binding, and mice conditionally deficient in Muc5b showed enhanced eosinophilic inflammation in response to airway instillation of IL-13, further validating that Muc5b carries glycan ligands for Siglec-F and suggesting that only subsets of airway mucins display the glycan structures necessary for Siglec-F binding (38). Although less is known about Siglec-8 tissue ligands, a recent study showed that Siglec-8 ligands were expressed selectively on serous cells, a subpopulation of submucosal gland cells in the inferior turbinate, and inflammation that occurs in chronic sinusitis led to increased expression of Siglec-8 tissue ligands in the upper airways (69). Additional studies show that mouse airways do not express Siglec-8 ligands and Siglec-8-Fc binding in human tracheal sections is restricted to serous cells in submucosal glands and cartilage (59) (Figure 4). The exact identity of these ligands is still under investigation, but given the fact that the galactose 6-*O*-sulfotransferase CHST-1 is dispensable for generating Siglec-F ligands in the mouse, it appears that the 6′ sulfation needed for Siglec-8 binding is not required for Siglec-F binding (70).

## Endocytosis of Siglec-F and Siglec-8

Beyond the physiological role of Siglec-8 in inducing cell death of eosinophils, Siglec-8 represents a promising target through which to deliver therapeutic payloads into eosinophils and other Siglec-8-expressing cells. Several studies have shown that siglecs are endocytic receptors and, once engaged, can carry their ligand—and presumably any associated cargo—into the cell (71–74). This strategy has been employed in the development of cancer therapeutics by targeting preferentially upregulated receptors such as the receptors for transferrin or folate or through antibodies targeting slightly more selectively expressed antigens, such as CD33 (Siglec-3) in acute myelogenous leukemia (75–77). An antibody-targeting CD22 (Siglec-2) is also under investigation to treat diseases involving B cells, such as B-cell acute lymphoblastic leukemia or systemic lupus erythematosus (78, 79). Due to the restricted expression pattern of Siglec-8, targeting of eosinophils through Siglec-8 offers promise. However, the capability to exploit Siglec-8 in this manner depends on whether Siglec-8 is endocytosed and is present and accessible on the surface of eosinophils in various circumstances. Siglec-F endocytosis has been studied in mouse eosinophils. Siglec-F is internalized in response to antibody ligation *via* a clathrin- and lipid raft-independent pathway that relies on ARF6 but not dynamin-1 (72). New data indicate that Siglec-8 is indeed internalized in response to antibody or synthetic ligand engagement on peripheral blood eosinophils and that this pathway can be exploited to deliver a toxin (the ribosome-inhibiting protein saporin) to eosinophils to induce cell death under conditions in which Siglec-8 engagement alone would be insufficient (i.e., in the absence of IL-5 priming) (80). Despite some similarities, including the lysosomal localization of the internalized siglec, the pathway utilized by Siglec-F internalization appears to be distinct from that of Siglec-8. The pathway of internalization can have profound effects on receptor function, leading to distinct signaling mechanisms and downstream functions or alterations in receptor turnover. For example, endocytosis of SR-A *via* a lipid raft/caveolae-dependent pathway is required for macrophage apoptosis in a ligand-dependent manner, whereas clathrin-mediated SR-A endocytosis is expendable for this effect (81). While there is abundant evidence linking endocytosis to the organization of signaling events (82), it remains to be determined whether the endocytosis of Siglec-8 affects its function.

Siglec-8 may also achieve part of its function by internalizing other surface proteins. Upon antigen stimulation, the B cell receptor (BCR) engages clathrin in lipid raft domains and thus is internalized *via* a mixed pathway (83, 84). While the siglec CD22 is initially excluded from lipid rafts, it colocalizes with the BCR and promotes its internalization when unmasked (85, 86). This downregulation of the BCR is thought to be one mechanism underlying the inhibitory function of CD22. Of note, the IL-5 receptor, which is critically important to the activation and survival of eosinophils, has been found to be internalized *via* distinct clathrin- and lipid raft-dependent pathways and is targeted for proteolytic degradation through the lipid raft-mediated endocytic pathway (87). It is an intriguing possibility that the endocytosis and trafficking of Siglec-8 and the IL-5 receptor may be linked in a way that influences the function of each receptor.

## Other Siglecs Found on Mouse and Human Eosinophils

While Siglec-8 has garnered much attention as a cell-surface marker of eosinophils, there are a number of other glycans and glycan-binding proteins present on eosinophils that regulate their survival, trafficking, and adhesion and that may be useful markers of eosinophilic inflammation. In both mice and humans, eosinophils express siglec family members other than Siglec-F and Siglec-8, respectively.

### CD22, Siglec-E, Siglec-G, and Mouse Eosinophils

Interestingly, mouse eosinophils in the gastrointestinal tract were found to express the siglec CD22 on their cell surface, a siglec previously thought to be restricted to B cells (88). CD22 expression was highest on eosinophils in the jejunum, although it was also found on eosinophils in the stomach, duodenum, or ileum. CD22 was not found on eosinophils in the blood or other tissues. The function of CD22 on these eosinophils is not yet clear, although CD22 ablation led to an increase in eosinophils in the jejunum, an effect that did not appear to be due to increased eosinophil differentiation from hematopoietic precursors or augmented IL-5 or eotaxin-2 signaling. In studies of mice overexpressing IL-5, which gives rise to eosinophilia, it was found that these eosinophils expressed mRNA for Siglec-E (orthologous to Siglec-9 in humans) and Siglec-G (orthologous to Siglec-10 in humans) (53). However, the surface expression and function of these siglecs on mouse eosinophils have not been studied.

### CD33, Siglec-7, Siglec-10, and Human Eosinophils

Immature human eosinophils express low levels of CD33 (Siglec-3) and downregulate this receptor upon maturation (89). Human eosinophils also express modest levels of Siglec-7 both in the peripheral blood and in nasal polyps (90, 91). In addition, Siglec-10, which was identified by four different groups through genomic analysis and screens of cDNA libraries (including one from asthmatic eosinophils), is expressed by human eosinophils (92–95). Siglec-10 possesses three tyrosine-containing cytoplasmic motifs—a membrane-proximal Grb2 binding motif, a central ITIM, and a membrane-distal ITSM or ITIM-like motif—and has been found to interact with SHP-1 and SHP-2 but not with SLAM-associated protein (48, 94). While Siglec-10 was detected on B cells using polyclonal antibody (93), a mAb detected Siglec-10 expression only on eosinophils, neutrophils, and monocytes and failed to detect expression on B cells (94), suggesting either that the polyclonal antibody was not specific to Siglec-10 (perhaps binding to another siglec family member present on B cells) or that a unique variant of Siglec-10 is expressed on these cell populations and not on B cells. Interactions between Siglec-10 and CD24 (heat stable antigen) (96), vascular adhesion protein-1 (97), and CD52 (98) have been demonstrated *in vitro* or on other cell types; however, the function of Siglec-10 in eosinophils has not been described. Indeed, little is known about the functions of any of these three siglecs on human eosinophils. Antibody ligation of Siglec-7 on eosinophils failed to induce apoptosis or prevent chemotaxis under conditions in which Siglec-8 ligation produced these effects (91), and the role of CD33 on eosinophils has similarly not been determined. Given the lack of functional data and the broader cell expression patterns for these siglecs, there has been less interest in exploiting these receptors to address eosinophilic inflammation.

## Selectins and Selectin Ligands on Eosinophils

Protein–glycan interactions are exceptionally important in the processes of cell adhesion and trafficking. In the initial steps of leukocyte extravasation, the cell must tether to and roll along the endothelium, which requires the interaction between selectins and their glycan ligands. Eosinophils depend to differing degrees on P-, E-, and L-selectin interactions in this process. Relative to neutrophils, eosinophils bind less well to E-selectin and bind to a greater extent to P-selectin through cell-surface glycan ligands (99), presumably due to increased levels of P-selectin glycoprotein ligand (PSGL)-1 (100). This same study found that L-selectin on the surface of eosinophils was important in tethering of eosinophils to human umbilical vein endothelial cells (HUVECs) but only due to establishing inter-eosinophil interactions rather than binding to the endothelial cells (99). However, others have found that diminished L-selectin expression or the use of blocking antibodies to L-selectin reduce eosinophil rolling and adhesion on HUVECs or on rabbit mesenteric venule endothelial cells under conditions of flow (101, 102). As demonstrated by the study by Sriramarao et al., human selectins are capable of interacting with ligands expressed in other species. Indeed, the selectin-binding sites in the best characterized P-selectin ligand, PSGL-1, are evolutionarily well conserved (103). However, distinct patterns of expression render cross-species comparisons more difficult. For example, P-selectin expression in mice but not in humans is induced by TNF-α or LPS, and cytokine regulation of human and primate P- and E-selectins is more selective than in mice (104, 105). However, mouse strains in which selectins have been knocked out have permitted elegant studies of their importance in mouse eosinophil migration. Using these mouse strains, several studies have shown that P-selectin is critical in eosinophil recruitment to the lung and peritoneum (106–108).

P-selectin glycoprotein ligand-1 is the best characterized P-selectin ligand on eosinophils, and its expression has been shown to be increased in patients with allergic asthma relative to healthy controls. This increase results in enhanced binding to P-selectin and IL-4-treated HUVECs (109). PSGL-1 contributes to eosinophil, but not neutrophil, adhesion to IL-13-activated HUVECs under conditions of physiological flow (110). Paradoxically, PSGL-1 expression is reduced on activated eosinophils and is shed from leukocytes (111). Although there have been reports that PSGL-1 can act as a ligand for E-selectin as well (112, 113), no changes in E-selectin binding were observed with eosinophils from allergic asthma patients in this study. There appear to be other significant ligands for P-selectin on eosinophils, however. In patients with atopic dermatitis, eosinophils are capable of binding substantially more soluble P-selectin than eosinophils from healthy donors but do not display more PSGL-1 on their surface (114).

Although the selectins bind to related sialylated glycans, these glycans can be present on a variety of different proteins or lipids that may be cell type- or tissue-specific. Eosinophils, for example, display far less sialyl Lewis^X^ antigen, a selectin ligand carbohydrate structure, than neutrophils, but a greater proportion is in the form of sialyl dimeric Lewis^X^ and is sensitive to endo-β-galactosidase treatment (115). Interestingly, neither the display of sialyl Lewis^X^ on eosinophils nor adhesion to immobilized E-selectin is protease-sensitive, indicating that these carbohydrate ligands may not be present on cell-surface proteins (115). Consistent with this, glycosphingolipids extracted from leukocytes were found to interact with E-selectin, and their biosynthesis was required for E-selectin-dependent, but not P-selectin-dependent, neutrophil adhesion (116). P-selectin ligands present on eosinophils clearly differ from those of E-selectin in that binding to immobilized P-selectin is protease-sensitive and endo-β-galactosidase-resistant (117). Indeed, although the expression of sialyl Lewis^X^ on eosinophils is not changed by cellular activation with platelet-activating factor (PAF), P-selectin binding is reduced following PAF activation and L-selectin is shed following transendothelial migration (117, 118).

Due to their critical role in eosinophil trafficking to peripheral tissues, selectins represent a potentially useful therapeutic target for diseases of eosinophilic inflammation. In fact, selectin antagonists interfere with eosinophil (and neutrophil) adhesion (119), and a pan-selectin antagonist glycomimetic agent is in clinical trials to modulate selectin-based adhesion in acute sickle cell crisis (120). Although lack of cell specificity is a concern, a more selective P-selectin antagonist or one that interacts with a eosinophil-selective P-selectin ligand would likely be effective in preventing further eosinophilic inflammation in the tissues with fewer potential complications.

## Galectin Family Members and Their Glycan Ligands on Eosinophils

The galectin family of proteins, previously known as S-type lectins, has a binding preference, generally, for β-galactosides, although there appear to be exceptions to this. Most of the members of the family are secreted but can cross-link cell-surface receptors due to the presence of more than one carbohydrate recognition domain (CRD) or through multimerization of a monomer containing one CRD (galectin-3).

The galectin family member most commonly associated with eosinophils is galectin-10, also known as Charcot–Leyden crystal (CLC) protein. Galectin-10 makes up about 10% of the total protein content of human eosinophils (121), and CLC deposition in tissues has long been considered a marker of eosinophilic (or basophilic) inflammation (122). The protein localizes to both the cytosol and a subset of core-less granules (123). The CLC protein was initially believed to function as a lysophospholipase within the eosinophil (121); however, this enzymatic activity has since been ascribed not the CLC protein but to another enzyme that can associate to a degree with it (124). Due to sequence identity, structural homology, and genomic structure, CLC protein became known also as galectin-10 (125–127). Unlike other members of the galectin family, however, galectin-10 appears not to bind β-galactosides to any substantial degree but instead appears to bind to mannose-containing carbohydrate moieties (128). The natural ligand or ligands of galectin-10 and the functional significance of its ability to bind to carbohydrates remain undetermined. Despite the lack of functional data, galectin-10 mRNA and protein levels remain useful biomarkers for eosinophilic airway inflammation, active eosinophilic esophagitis, aspirin-exacerbated respiratory disease, CRTH2 activation, and celiac disease (129–133).

On the eosinophil cell surface, galectin–ligand interactions have been found to be important in eosinophil recruitment, activation, and survival. The granulocyte-specific and heavily glycosylated protein CD66b (also known as CEACAM8) is expressed on eosinophils and is upregulated in response to cellular activation (134, 135). Sialylated glycans on CD66b interact with E-selectin, and this interaction has been shown to be important for neutrophil adherence to activated endothelium (136). Glycans on CD66b also interact with galectin-3, and engagement of CD66b using either soluble galectin-3 or antibody induced ROS production and degranulation (137). Cross-linking of CD66b also caused the eosinophils to become more adherent, perhaps through the clustering of the integrin subunit CD11b.

Several other galectin interactions may be important in eosinophil adhesion and chemoattraction, including those with galectins-1, -3, and -9 (138–140). However, the role of galectins in eosinophil recruitment is covered in greater detail in a review in this volume by Sriramarao et al. and will not be discussed further here. It should be noted, however, that galectins play other important roles in eosinophil biology and are biomarkers of disease activity. High concentrations of galectin-1, for example, can induce eosinophil cell death (140), and levels of galectin-3 before treatment in patients with severe asthma predict treatment responses to omalizumab (141).

## Eosinophil Glycomics

While many of the cell-surface and intracellular eosinophil proteins have been identified and extensively described, the glycans that coat the various cell-associated proteins and likely play important roles in numerous biological pathways remain largely shrouded in mystery. As part of an effort to characterize these glycans, the glycome of human eosinophils has been analyzed in cell lysates and compared to those of basophils and mast cells to elucidate the identities of these glycans, their relative abundances, and key differences between these cell types (142). Although mast cells possess substantial amounts of terminally sialylated epitopes on their various glycoproteins, eosinophils and basophils have far more part-processed terminal *N*-acetylglucosamine (GlcNAc)-containing structures. For example, the most abundant *N*-glycan by far in both eosinophils and basophils is a bi-antennary structure with two terminal non-extended GlcNAc sugars, which is far less abundant in mast cells. While the functional relevance of these patterns is unclear, it is unlikely that these modifications to the cell surface are random. In addition, it is uncertain how cytokine priming and other signals that may be present under inflammatory conditions may affect glycan synthesis and processing. However, such changes may well affect processes such as adhesion, activation, cell–cell interaction, and even survival.

## Conclusion

There remains a clinical need to effectively and selectively treat diseases of eosinophilic inflammation. Due to their roles in recruitment, adhesion, activation, and survival, glycan–glycan-binding protein interactions are beginning to garner attention as therapeutic prospects. Siglec-8 and its ligands offer a cell-selective pathway to induce cell death in primed eosinophils and deliver therapeutic payloads into the cell. Antagonists of selectin interactions may help limit eosinophilic inflammation, but significant hurdles remain for achieving a safe, cell-selective effect. Monomeric β-galactosides or glycomimetics may also be clinically useful in antagonizing eosinophil lectin interactions that are involved in cell adhesion and activation. While targets and biomarkers have been identified, further studies are necessary to elucidate the functions of glycan-binding proteins on eosinophils, such as those of other members of the siglec family; to identify their natural glycan ligands and how they are modulated; and to determine the functional significance of the glycans displayed on the eosinophil cell surface.

## Author Contributions

JO completed the sections on Siglec-8 endocytosis, selectins, galectins, and other members of the siglec family. DC completed all other sections regarding Siglec-8 as well as Siglec-F. BB organized the effort and wrote the introduction. All authors contributed to revisions of the manuscript.

## Conflict of Interest Statement

BB has current or recent consulting or scientific advisory board arrangements with or has received honoraria from, Sanofi-Aventis, TEVA, AstraZeneca and Allakos, and owns stock in Allakos and Glycomimetics. He receives publication-related royalty payments from Elsevier and UpToDate™ and is a co-inventor on existing Siglec-8-related patents and thus may be entitled to a share of royalties received by Johns Hopkins University on the potential sales of such products. BB is also a co-founder of Allakos, which makes him subject to certain restrictions under University policy. The terms of this arrangement are being managed by the Johns Hopkins University and Northwestern University in accordance with their conflict of interest policies. The authors have no additional competing financial interests.
